# The Nurses' Enabling Bill

**Published:** 1919-11-29

**Authors:** 


### THE MATRONS' AND SISTERS' DEPARTMENT.

### THE NURSES' ENABLING BILL.

SOME considerable experience of the manner in which the House of Commons has hitherto grappled with the subject of registration for nurses has made it clear that, to use the words of Dr. Addison, they "are not competent to settle controversies as to the representation of this or that body." These matters are not "appropriate topics for discussion" in Parliament. They need consideration by a much smaller body, one acquainted with facts, and not dependent on rhetorical statements. For this reason the Bill for the State Registration of Nurses, so universally acclaimed on its second reading in the House of Commons, goes little farther than conferring on sixteen nurses and nine other persons the duty of drawing up a scheme under which nurses may obtain State recognition. The Bill, in fact, *enables* nurses to put their house in order under the ægis of the State. It procures for the nursing profession what it is proposed to procure for the Church under the famous Enabling Bill. It throws on the profession the onus of self-regulation.

It is a large liberty of choice which is thus acquired. It devolves heavy responsibilities on those selected to form the first Council under the Act. It throws a still heavier responsibility on the Minister who must select the first sixteen nurses who are to co-operate with the first nine representatives of the Privy Council, the Board of Education, and the Minister of Health, in framing the outlines of the measure. But the danger of the nursing profession being betrayed by any lowering of standard or reckless interference with good work now under accomplishment is almost negligible. There is a practical consensus of opinion as to the best methods of training nurses. And should anything be proposed which the main body of nurses believed to be subversive of their interests, it could hardly escape deletion in the subsequent scrutiny to which all the regulations will be subjected before being ratified. Nurses will be in a clear majority on the Council from the beginning and always, and this is the safeguard they ardently desire.

We note with great interest that, although the Bill is merely a skeleton, powers are conferred on the Council to render the register popular, and this is of all things essential if it is to be no mere dead letter. The Council will be able to make provision "with respect to the uniform or badge which may be worn by nurses so registered." It is an age of badges, and few of us take the trouble to examine very carefully what kind of badge our neighbour chooses to wear. But it is also an age of uniform, and the uniform, if it be really distinctive, hits the eye of the least observant. If registered nurses obtain the privilege of a protected uniform, little doubt but that all but a negligible few will enter their names on the register.

There is no doubt that a period of doubt and prejudice will have to be overcome in respect to registration. No sooner is it an accomplished fact than we may expect our old friend, "What is the good of it?" to arrive at the gates trying to keep back the entering crowds. Some of the nurses, with certificates which ensure them whatever they want, will pour scorn upon a register which during two years must admit the names of many less well equipped for their duties than themselves. A party will be formed against registration should it fail instantaneously to secure for all nurses higher remuneration and shorter hours. These obstacles are to be expected. The way to counter them is to supply registered nurses with some tangible undeniable *cachet*. We want something which will appeal not only to the "wise women of nursing," but to the foolish also. After all, have we not all a foolish side? Let us realise that there is often sound value in the simple, instinctive demands of people who have not reasoned very profoundly, but know their own minds in one particular.

Nurses are agreed in their desire for a uniform which cannot be copied by their inferiors or by the base. If to this concession to popular and just feeling there could be added the concession of some less ambiguous title than *nurse*, now appropriated by maidservants as well as by the untrained, the victory, so far as making registration universal, would be won. As sole users of the title, Nursing Sister, distinguished wherever they went by their distinctive uniform, the registered would number the entire profession. Now be it remembered that hitherto wherever a system of voluntary State registration has been tried the registered have been found to number merely a minority of the trained nurses eligible for registration, and consequently registration has been a failure. Let us be warned in time.

